# Plasma Membrane Ca^2+^ Permeable Mechanosensitive Channel *OsDMT1* Is Involved in Regulation of Plant Architecture and Ion Homeostasis in Rice

**DOI:** 10.3390/ijms21031097

**Published:** 2020-02-07

**Authors:** Jiayan Liang, Yan He, Qiuxin Zhang, Wenyi Wang, Zemin Zhang

**Affiliations:** State Key Laboratory for Conservation and Utilization of Subtropical Agro-Bioresources, Guangdong Provincial Key Laboratory of Plant Molecular Breeding, South China Agricultural University, Guangzhou 510642, China; me_ljy@126.com (J.L.); hy9509@126.com (Y.H.); qxzhang1993@163.com (Q.Z.)

**Keywords:** rice, plant architecture, dwarf and multi-tillering1 (*dmt1*), ion homeostasis, Ca^2+^ sensors

## Abstract

Plant architecture is an important factor for crop production. Plant height, tiller pattern, and panicle morphology are decisive factors for high grain yield in rice. Here, we isolated and characterized a T-DNA insertion rice mutant *Osdmt1* (Oryza sativa dwarf and multi-tillering1) that exhibited a severe dwarf phenotype and multi-tillering. Molecular cloning revealed that *DMT1* encodes a plasma membrane protein that was identified as a putative Ca^2+^ permeable mechanosensitive channel. The transcript expression level was significantly higher in the *dmt1* mutant compared to wild type (WT). Additionally, the *dmt1* homozygous mutant displayed a stronger phenotype than that of the WT and heterozygous seedlings after gibberellic acid (GA) treatment. RNA-seq and iTRAQ-based proteome analyses were performed between the *dmt1* mutant and WT. The transcriptome profile revealed that several genes involved in GA and strigolactone (SL) biosyntheses were altered in the *dmt1* mutant. Ca^2+^ and other ion concentrations were significantly enhanced in the *dmt1* mutant, suggesting that *DMT1* contributes to the accumulation of several ions in rice. Moreover, several EF-hand Ca^2+^ sensors, including CMLs (CaM-like proteins) and CDPKs (calcium-dependent protein kinases), displayed markedly altered transcript expression and protein levels in the *dmt1* mutant. Overall, these findings aid in the elucidation of the multiply regulatory roles of *OsDMT1/OsMCA1* in rice.

## 1. Introduction

Plant architecture is a complex of crucial agronomic traits that determine grain yield. In rice, plant architecture is mainly determined by several factors, including plant height, branching pattern, and leaf and panicle morphologies [1,2]. Moderate plant height is an important basis for rice breeding, and the substantial increase in rice yield during the “green revolution” benefited from the recessive semi-dwarf gene 1 *(sd1)* [3]. A number of dwarf and semi-dwarf mutants have been reported and functionally isolated in rice. In particular, several *DWARF* genes, such as *D3*, *D10*, *D17*, and *D27*, have been proven to be involved in biosynthesis and signaling pathways of strigolactones (SLs), leading to a change in tiller number or/and plant height in rice [4,5,6,7]. Rice tiller number is considered to be one of the most crucial factors for ideal rice architecture because tiller number per plant determines the number of panicles, which is a key component of grain yield [8]. Several key quantitative trait loci (QTLs) and/or genes that regulate tiller bud formation and outgrowth were identified and functionally characterized in rice such as *MONOCULM1(MOC1)*, *MOC2*, and *MOC3* [8,9,10]. However, the mechanism underlying plant architecture is not completely understood.

Plant hormones are a group of naturally occurring substances, which play a prominent role in regulating plant development and signaling networks at quite low concentrations [11], including jasmonates (JA), cytokinins (CK), auxin (IAA), gibberellins (GA), abscisic acid (ABA), salicylic acid (SA), ethylene (ET), brassinosteroids (BR), and strigolactones(SL). Over the past decades, plant hormones, particularly GA, BR, and SL, have been proven to extensively participate in the regulation of plant architecture. GA and BR are two predominant hormones that determine plant height and leaf angle by regulating cell elongation [12,13,14,15]. Mutants that are deficient in GA or BR result in reduced plant growth and dwarfism. Although it has been known that GA and BR function redundantly in many developmental processes [16], emerging evidence shows that BR-GA crosstalk regulates plant growth and development [12,16]. The SLs comprise a group of terpenoid lactones that play important roles in the inhibition of shoot branching, leading to changes in tillering. Several SL biosynthesis and signaling components have been identified in various plant species, such as *MORE AXILLARY GROWTH (MAX)* in *Arabidopsis*, *DWARF (D)* in rice [17,18]. 

Ca^2+^ ions act as a vital second messenger in plant cells during various developmental processes and in response to environmental stimuli, including pathogens, hormones, and abiotic stresses. Plants have evolved a diversity of Ca^2+^-binding proteins (CBPs) that serve as Ca^2+^ sensors that bind to Ca^2+^ with the evolutionarily conserved EF-hand motif, which consists of helix-loop-helix structures [19,20]. In plants, the three largest categories of EF-hand Ca^2+^ sensors were identified, include CaMs (calmodulins), CMLs (CaM-like proteins) and CDPKs (calcium-dependent protein kinases) [21]. It is now well acknowledged that these Ca^2+^ sensors are extensively linked to hormone response and stress signaling. Notably, emerging evidence has also shown that Ca^2+^ is involved in various developmental processes, such as embryogenesis, seed germination, and flowering. For example, transgenic *Arabidopsis* plants with reduced expression of *CML24* exhibited resistance to ABA inhibition of germination and seedling growth; moreover, a defect in long-day induction of the transition to flowering was detected in *CML24*-underexpressing transgenic *Arabidopsis* plants [22].

Herein, to identify genes regulating plant architecture in rice, a *dwarf and multi-tillering1 (dmt1)* mutant was isolated and characterized by T-DNA insertion lines with Zhonghua 11 (*japonica*) background. The *dmt1* mutant exhibited severely defective plant architecture, such as dwarfism, high tiller numbers, and decreased grain length and width. The *DMT1* gene encodes a plasma membrane protein, OsMCA1. Transcriptome and proteome profiles revealed that GA and SL biosyntheses were altered in the *dmt1* mutant; moreover, EF-hand Ca^2+^ sensors, including CMLs (CaM-like proteins) and CDPKs (calcium-dependent protein kinases) were also markedly altered in transcript expression and protein levels in the *dmt1* mutant. Further study revealed that the Ca^2+^ concentration was significantly enhanced in the *dmt1* mutant than in the wild type (WT), indicating that *OsDMT1* was potentially involved in ion transport or/and accumulation in rice. Taken together, the present study provides a better understanding of the mechanisms underlying the plant architecture of rice.

## 2. Results

### 2.1. Rice dmt1 Mutant Exhibits Sever Dwarf and More Tillers

**Figure 1:**
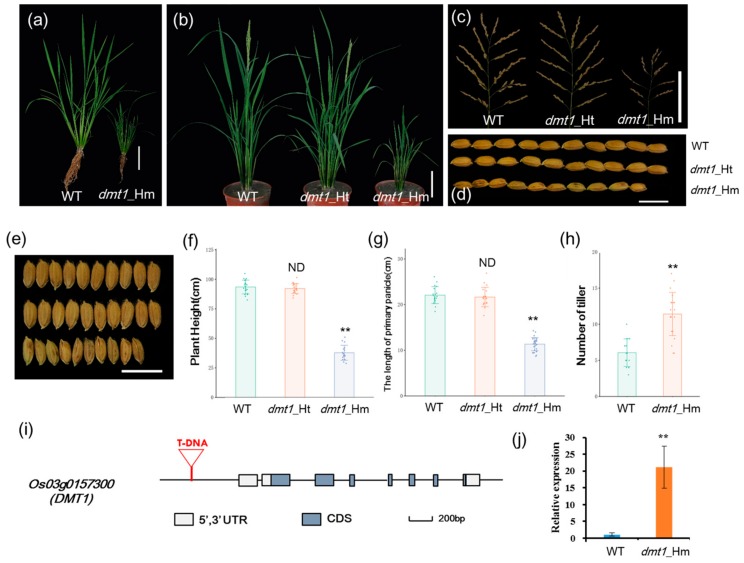
Characterization of the rice dwarf and multi-tillering1 *(dmt1)* mutant plants. (**a**) *dmt1* homozygous mutant exhibited a dwarf phenotype in the tillering stage. Scale bar: 10 cm. (**b**) *dmt1* homozygous mutants exhibited dwarf and multi-tillering phenotypes compared with those of the wild type (WT) and heterozygous mutants during the reproductive stage. Scale bar: 10 cm. (**c**) The phenotype of panicle branching in the WT (left), *dmt1* heterozygous mutant (middle), and *dmt1* homozygous mutant (right). Scale bar: 10 cm. (**d**,**e**) Seed length and width in the WT (upper), *dmt1* heterozygous mutant (middle), and *dmt1* homozygous mutant (bottom). Scale bar: 1 cm. (**f**,**g**) Statistical analysis of plant height (**f**) and primary panicle length (**g**) in the WT, *dmt1* heterozygous mutant, and *dmt1* homozygous mutant. Values represented by means ± SE. ∗∗ *p* < 0.01, Student’s *t*-test, *n* = 30; (**h**) Statistical analysis of the number of tillers in the WT and *dmt1* homozygous mutant. Values are expressed is represented by means ± SE. ∗∗ *p* < 0.01, Student’s *t*-test; *n* = 30. (**i**) Schematic diagram of *DMT1*. *DMT1* consisted of eight exons and seven introns. T-DNA inserted into the promoter region. Boxes and lines represent exons and introns, respectively. (**j**) *DMT1* transcripts were detected in the *dmt1* mutant and the WT using qRT-PCR. *ACTIN1* was used for a control. ** *p* < 0.01, Student’s *t*-test.

To identify new regulators of plant architecture in rice, a dwarf and multi-tillering1 *(dmt1)* mutant was isolated from a T-DNA insertion population with Zhonghua 11 (*japonica*) background. The *dmt1* mutant displayed a severe dwarf phenotype during the tillering and reproductive stages (Figure 1a,b). The plant height and length of the primary panicle of the homozygous *dmt1* mutant were approximately 59.5% and 48.6% lower than those of the WT, respectively (Figure 1c,f,g). Additionally, other phenotypic differences were observed between the *dmt1* mutant and the WT, such as grain length and width, effective panicles, and spikelet number per panicle (Figure 1d,e; Figure S1). Notably, an inverse effect between plant height and tiller number was detected in the *dmt1* mutant, namely, *dmt1* produced more tillers than that by the WT (Figure 1h). Overall, the results indicated that the mutation of *dmt1* led to abnormal development in rice, particularly in the plant architecture, including dwarfism and a greater number of tillers.

Hygromycin resistance test showed that the *dmt1* cosegregate with the resistance maker in the T_3_ population. Of 517 T_3_ individuals, the progeny of plants exhibited phenotypic segregation of normal height to dwarf of ~3:1 (389:128), indicating that the mutant phenotype was controlled by a single recessive gene. To isolate the gene disrupted in the *dmt1* mutant, Inverse polymerase chain reaction (IPCR) was used to separate the T-DNA flanking region [23]. Sequence analysis revealed that T-DNA was inserted in 1949 bp upstream of the start codon *Os03g0157300* (Figure S2), which was predicted to be a plasma membrane protein *OsMCA1 (Mid1-complementing activity1)*. *Os03g0157300* was potentially the gene responsible for the *dmt1* mutant phenotype and was designated as *DWARF AND MULTI-TILLERING1 (DMT1)*. The *OsDMT1* gene consisted of eight exons and seven introns (Figure 1i). qRT-PCR was performed to examine *DMT1* transcript levels in the mutant and WT. The result showed that the expression of *DMT1* was significantly higher (~21 fold) in the *dmt1* mutant than in the WT (Figure 1j). 

### 2.2. Transcriptome and Proteomic Analysis of the dmt1 Mutant

To investigate the molecular basis of the phenotype of the *dmt1* mutant and the WT, the RNA expression profiles of the *dmt1* mutant and WT were analyzed and compared in three biological replicates. Moreover, owing to protein abundance levels that were not always consistent with mRNA expression levels, the iTRAQ-based proteome profile was also identified in the *dmt1* mutant and WT to provide a deeper insight into post-transcriptional modifications. Because the most obvious phenotypic differences were observed during the tillering stage, the transcriptome and proteome were analyzed in the *dmt1* mutant and WT at the tillering stage. In total, 33,746 genes and 3663 proteins were detected using RNA-seq and iTRAQ, respectively (Tables S2 and S3). Differentially expressed genes (DEGs) were determined using the DESeq2 package with the criteria of *q* value < 0.005 and |log2 (fold change) |> 1 set as thresholds. For protein quantitative analysis, fold changes >1.5 and <0.67 were set to differentially abundant proteins (DAPs) with up- and down-regulated, respectively, with a *q* value < 0.05. Notably, the *DMT1* gene displayed significantly upregulated in *dmt1* mutant compared to WT in RNA-seq (Table S4), which consistent with qRT-PCR result (Figure 1j).

**Figure 2:**
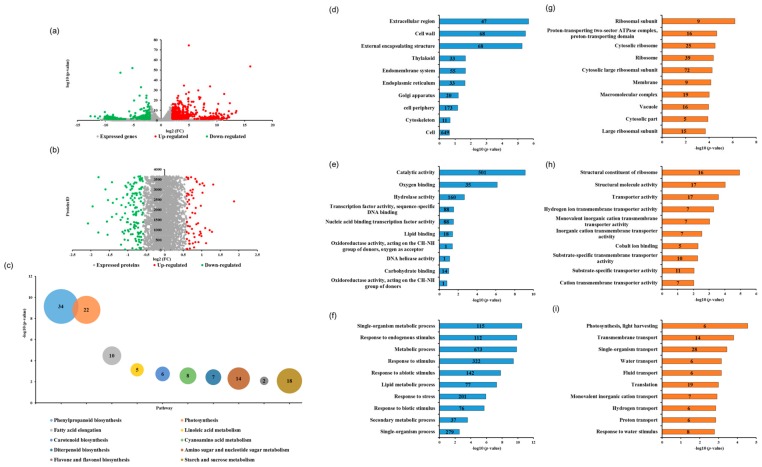
Transcriptome and proteomic analysis of the *dmt1* mutant and WT. (**a**) Volcano plot of genes that were differentially expressed between the *dmt1* mutant and WT in RNA-seq (1069 upregulated and 235 downregulated). The red dots in the right of the plot represent significantly upregulated genes, and the green dots in the left of the plot represent significantly downregulated genes. (**b**) Differentially abundant proteins (DAPs) in iTRAQ. Red spots indicate 43 upregulated proteins, and green spots indicate 104 downregulated proteins in the *dmt1* mutant compared to the WT. (**c**) Top 10 pathway enrichments of DEGs, the ordinate indicates -log_10_ (*p*-value), bubble size and date labels indicate gene numbers. (**d**–**f**) Top 10 GO ontology enrichment of DEGs based on cellular component (**d**), molecular function (**e**), and biological process (**f**). The abscissa indicates -log10 (*p*-value), and the number in the data labels indicate the gene numbers. (**g–i**) Top-10 GO ontology enrichment of DAPs based on cellular component (**g**), molecular function (**h**), and biological process (**i**). The abscissa indicates -log10 (*p*-value), the number in the data labels indicate gene numbers.

Global analysis of the correlation between transcriptome and proteome data was performed between *dmt1* mutant and WT (Figure S3). Among all identified genes and protein species, 1304 DEGs (1069 upregulated and 235 downregulated) and 147 DAPs (43 upregulated and 104 downregulated) were identified (Figure 2a,b). Overall, the number of upregulated DEGs was greater than that of downregulated ones, whereas the number of upregulated DAPs was lower than the number of downregulated ones, suggesting that posttranscriptional regulation affected the abundance of protein species. To investigate the functions of DEGs, pathway enrichment analysis was performed based on the Kyoto Encyclopedia of Genes and Genomes (KEGG) pathway, results showed the significantly enriched pathways were related to several metabolic pathways, such as phenylpropanoid biosynthesis, starch and sucrose metabolism and photosynthesis in *dmt1* mutant compared to WT (Figure 2c). To further obtain the insight of the functional categories, the identified DEGs and DAPs were classified into different groups based on cellular component, molecular function, and biological process (Figure 2d–i). According to the Gene Ontology (GO) terms of the cellular component, the major categories included extracellular region, cell wall and external encapsulating structure (Figure 2d). As for the Gene Ontology enrichment analyses, results showed that single-organism metabolic process, response to endogenous stimulus, and metabolic process were the major categories annotated for the biological process (Figure 2f). Additionally, proteomic analysis revealed that DAPs were mostly involved in transmembrane transport, single-organism transport (Figure 2i). 

### 2.3. OsDMT1 Involved in GA Metabolism

**Figure 3:**
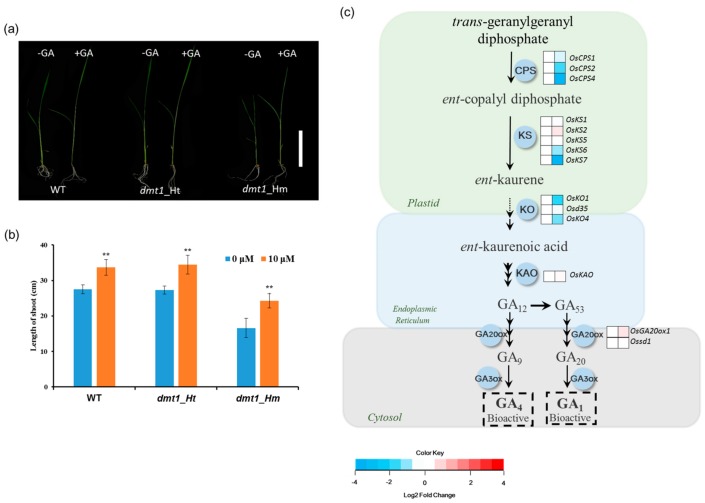
*OsDMT1* involved in GA metabolism (**a**) GA response in the WT (left), heterozygous mutant (middle), and homozygous mutant (right). The 10-day-old WT, *dmt1* homozygous, and heterozygous seedlings were sprayed with 0 μM or 10 μM gibberellin three times every 2 days, and the lengths of the shoots were measured on the 7^th^ day after the first treatment. Scale bar: 10 cm. (**b**) Quantitative measurements of the lengths of the shoot after GA treatment on the 7^th^ day in the WT, *dmt1* heterozygous mutant and homozygous mutant. Values represented by means ± SE. ∗∗ *p* < 0.01, Student’s *t*-test; (**c**) Gene expression in the GA biosynthesis pathway between the *dmt1* mutant and the WT. In the heatmap for each gene, two cells represent the WT and *dmt1* mutant (left to right). Gene expression levels in the *dmt1* mutant are indicated in the heatmaps by the log2 fold change relative to that of the WT. The dashed arrow represents several enzymatic reactions. CPS: ent-copalyl diphosphate synthase; KS: ent-kaurene synthase; KO: ent-kaurene oxidase; KAO: ent-kaurenoic acid oxidase; GA20ox: gibberellin 20-oxidase; GA3ox: gibberellin 3-oxidase; IPT: adenylate dimethylallyltransferase.

GA, an important phytohormone stimulating plant growth and development, plays an important role in determining plant height [24,25]. Because the *dmt1* mutant displayed a severe dwarf phenotype during the tillering and heading stages, it encouraged us to explore whether the *dmt1* mutant is involved in GA biosynthesis. To examine the *dmt1* mutant response to GA, 10-day-old WT, *dmt1* homozygous, and heterozygous seedlings were sprayed with either 0 μM or 10 μM gibberellin, the results showed that the length of shoots increased after exogenous GA treatment in WT, homozygous, and heterozygous seedlings (Figure 3a). Notably, the *dmt1* mutant displayed a stronger phenotype compared with that of the WT and heterozygous seedlings after GA treatment The length of shoots in the WT increased by 1.15-fold, whereas the increase was 1.30-fold in the *dmt1* homozygous mutant after GA treatment (Figure 3b), a more detailed analysis, such as GA level quantitation is required to build a robust link between DMT1 and GA biosynthesis. 

Given that the *dmt1* mutant was potentially involved in the GA metabolism in rice, we systematically analyzed the expression of GA biosynthesis genes using the RNA-seq dataset. GA biosynthesis starts with geranyl geranyl diphosphate which is converted to *ent*-kaurene by the action of CPS (*ent*-copalyl diphosphate synthase) and KS (*ent*-kaurene synthase) in the plastid. Several *CPS* and *KS* genes displayed significantly downregulated in the *dmt1* mutant, such as *OsCPS4* and *OsKS7* (Figure 3c, Table S5). KO (*ent*-kaurene oxidase) and KAO (*ent*-kaurenoic acid oxidase) are two key enzymes to convert ent-kaurene to GA_12_, which considered as the common precursor of all GAs in plants [26]. The transcript of *KO2* was more downregulated in the *dmt1* mutant than in the WT (Figure 3c, Table S5). These results indicate that *dmt1* was potentially involved in GA metabolism in rice.

### 2.4. DWARF 14 (D14) and D17, Two Strigolactones (SLs)-Related Genes, Displayed Decreased Expression in the dmt1 Mutant

**Figure 4:**
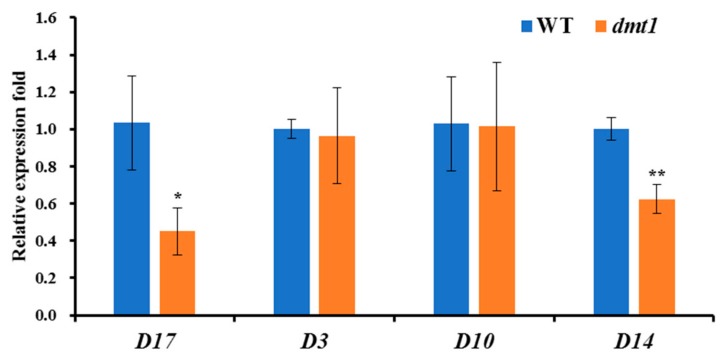
The expression patterns of *DWARF3 (D3)*, *D10*, *D14*, and *D17* between the *dmt1* mutant and the WT. *ACTIN1* was used for a control. * *p* < 0.05, ** *p* < 0.01, Student’s *t*-test.

Apart from the severe dwarf phenotype detected in the *dmt1* mutant, the number of tillers was higher in the *dmt1* mutant than in the WT. Strigolactones (SLs) are a class of important plant hormones regulating rice tillering [27]; thus, we examined the expression patters of several genes involved in SL biosynthesis and perception in rice, such as *DWARF3 (D3)*, *D10*, *D14*, and *D17*. As depicted in Figure 4, *D14* and *D17* were significantly lower in the *dmt1* mutant than in the WT, whereas *D3* and *D10* were not significantly different between the *dmt1* mutant and the WT (Figure 4), indicating that *D14* and *D17* are potentially involved in tiller formation in the *dmt1* mutant.

### 2.5. Several Ions Accumulated in the dmt1 Mutant

Considering that OsMCA1 is a plasma membrane protein and identified as a putative Ca^2+^ permeable mechanosensitive channel in *Arabidopsis* and rice [28], to investigate whether *DMT1* was involved in ion uptake in rice, Ca^2+^ concentrations between the *dmt1* mutant and the WT were measured. As depicted in Table 1, Ca^2+^ was significantly higher in the *dmt1* mutant than in the WT. We also measured the level of other ions, and the results showed that Mg^2+^, Mn^2+^, and Zn^2+^ were also significantly increased in the *dmt1* mutant, whereas there was no significant difference in Al^3+^ and Cu^2+^ (Table 1), suggesting that *DMT1* contributed to the accumulation several ions in rice.

### 2.6. Ca Sensor Genes Displayed Distinct Differential Expression in the dmt1 Mutant

**Figure 5:**
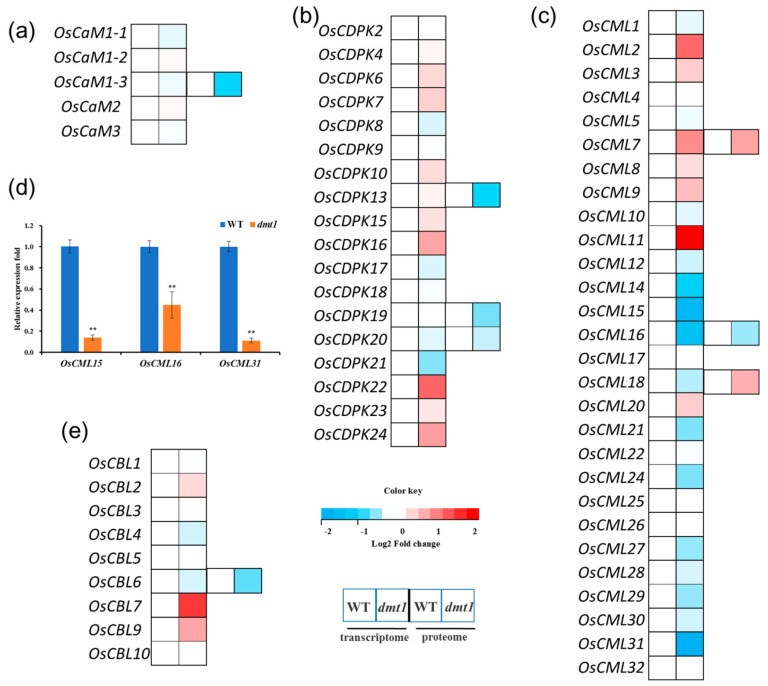
Expression patterns of Ca sensor genes and proteins between the *dmt1* mutant and the WT. (**a–c**) Expression patterns of CaMs (**a**), CDPKs (**b**), and CMLs (**c**) in the transcriptome and proteome. In the heatmap for each gene, the first two cells represented the WT and the *dmt1* mutant (left to right) within the transcriptome, and the third and fourth cells represented the WT and the *dmt1* mutant (left to right) within the proteome. (**d**) The expression of *OsCML15, OsCML16,* and *OsCML31* was validated by qRT-PCR; *ACTIN1* was used for a control, ** *p* < 0.01, Student’s *t*-test; (**e**) Expression patterns of CBLs in the transcriptome and proteome. In the heatmap for each gene, the first two cells represented the WT and the *dmt1* mutant (left to right) within the transcriptome, and the third and fourth cells represented the WT and the *dmt1* mutant (left to right) within the proteome.

In plants, three largest categories of EF-hand Ca^2+^ sensors are CaMs (calmodulins), CMLs (CaM-like proteins) and CDPKs (calcium-dependent protein kinase) [21]. The expression profiles of CaMs, CMLs, and CDPKs were examined with the RNA-seq dataset. The results showed that all five *OsCaMs* were not significantly different between the *dmt1* mutant and the WT (Figure 5a, Table S5). Similarly, the expression of most of *OsCDPKs* were not significantly different between the *dmt1* mutant and the WT (Figure 5b). However, several *OsCMLs* were significantly differentially expressed (either upregulated or downregulated) in the *dmt1* mutant (Figure 5c); for example, *OsCML2* and *OsCML11* were significantly higher in the *dmt1* mutant than in the WT, whereas some *OsCMLs,* such as *OsCML15* and *OsCML16* were significantly downregulated (Figure 5c, Table S5). The expression patterns of several OsCMLs were confirmed with qRT-PCR (Figure 5d). Several proteins were also detected in the *dmt1* mutant and the WT with iTRAQ in the proteome database, such as OsCaM1-3, OsCBL6, OsCDPK13, and OsCDPK19 (Figure 5a–c,e, Table S5). Generally, the protein level change trend was consistent with the transcript expression level. Furthermore, we analyzed the expression patterns of calcineurin B-like (CBL) proteins in the *dmt1* mutant. CBLs are a group of calcium sensors present in plants and believed to perceive fluctuations in the cellular calcium level [29]. The majority of *OsCBLs* were not significantly different between the *dmt1* mutant and the WT (Figure 5e). Overall, the above results suggested that *OsCMLs* might be involved in Ca^2+^ signal transduction in the *dmt1* mutant.

## 3. Discussion

Plant architecture directly regulates biomass in plants. Identification and characterization of mutants that displayed defects in plant architecture allowed us to elucidate the underlying molecular mechanisms [30]. In the present study, a mutant defect in plant height and tiller number termed *dwarf and multi-tillering1 (dmt1)* was isolated in T-DNA insertion lines in the rice cultivar Zhonghua 11 (ZH11). Transfer DNA (T-DNA) of *Agrobacterium tumefaciens* has been proven to be a successful and effective tool for gene identification in various plant species. The homozygous *dmt1* mutant displayed severe dwarfism and increased tiller number during the tillering and heading stages. Moreover, other phenotypic differences were observed between the *dmt1* mutant and the WT, such as grain length and width and number of effective panicles. Our results further showed that *DMT1* encodes a plasma membrane protein, MCA1 [31,32]. Kurusu et al. (2012a) revealed that *OsMCA1* is expressed throughout different developmental stages, and *OsMCA1*-suppressed lines exhibited slower growth in transgenic plants [31]. This was further examined by Liu et al. (2015), who reported that a single nucleotide polymorphism (SNP) in *OsMCA1* led to plant architecture defects in rice; moreover, the expression of *OsMCA1/PAD* was significantly decreased in young and mature leaves in the *pad* mutant, and *OsMCA1/PAD-*overexpressing plants do not exhibit an obvious phenotype in rice [32]. Notably, in our study, the expression of *OsDMT1* was significantly higher in the *dmt1* mutant than in the WT (Figure 1j), this might be due to the function of enhancer elements in the T-DNA region. Recently Several gain-of-function rice T-DNA insertion mutants were identified, such as *OsHKT1;4, OsDOF24, OsBG1* [33,34,35]. For example, OsHKT1;4, the T-DNA inserted approximately 3kb upstream of the initiation codon of OsHKT1;4, leading to increased expression of OsHKT1;4 and displayed lower concentrations of Na^+^ in the homozygous mutant young leave and stems [34]. In *Arabidopsis*, overexpression of *AtMCA1* caused severe developmental defects, including short stems, small rosettes, and no petals [28]. The above results indicate that *MCA1* regulates plant growth and development in a dose-dependent manner. To investigate the molecular basis of the phenotype of the *dmt1* mutant and the WT given that the most obvious phenotypic differences were detected in the tillering stage, an integrated omics approach combining the transcriptome and proteomics was performed between the *dmt1* mutant and the WT at the tillering stage. Totally, 1304 DEGs (1069 upregulated and 235 downregulated) and 147 DAPs (43 upregulated and 104 downregulated) were identified, respectively (Figure 2a,b). Functional analysis indicated that the abundance of DEGs was related to several metabolic processes, such as single-organism metabolic process, response to endogenous stimulus, and metabolic process, whereas abundance of DAPs was related to in transmembrane transport, single-organism transport. The expressions of several proteins involved in transmembrane transport were observed to markedly change in the proteomics profile. *DMT1*, which encodes a plasma membrane protein, was identified as a putative Ca^2+^ permeable mechanosensitive channel in plants. Alteration of *DMT1* leads to the regulation of transmembrane transport. Overall, the integrated omics approach contributed to elucidation of the mechanisms responsible for plant architecture in rice.

Considering that the *dmt1* mutant displayed a severe dwarf phenotype during the different developmental stages, we determined the response to GA between the *dmt1* mutant and the WT. We used GA to treat WT, *dmt1* homozygous, and *dmt1* heterozygous seedlings and longer shoots were detected in the *dmt1* homozygous compared to WT and heterozygous seedlings (Figure 3a,b). We further analyzed the expression of genes involved in GA biosynthesis, and the results showed that several key genes were downregulated in the *dmt1* mutant, such as *OsCPS2* and *OsKS6* (Figure 3c). Phytohormone GAs are biosynthesized from geranylgeranyl diphosphate (GGDP), convert to *ent*-kaurene in two steps via the CPS (ent-copalyl diphosphate synthase) and KS (ent-kaurene synthase) [36]. In rice, *OsCPS2* is contiguously clustered with three *KS*-like genes, namely, *OsKS5*, *OsKS6,* and *OsKS7* [36]. Both CPS and KS are involved in the initial step of bioactive GA biosynthesis, suggesting that the early step of GA biosynthesis was more regulated in the *dmt1* mutant than in the WT. We further explored the expression patterns of SL-related genes because the number of tillers was increased in the *dmt1* mutant. *DWARF14 (D14)* and *D17* displayed significantly lower *dmt1* than that in the WT, whereas *D3* and *D10* did not differ significantly in *dmt1* (Figure 4). Previous studies have shown that *D14* inhibited tillering and acted as an important component of the SL-dependent branching inhibition pathway in rice [37,38,39]. Taken together, the present results suggested that some genes involved in GA and SL biosynthesis might be altered in the *dmt1* mutant.

Calcium ions (Ca^2+^) play important roles during plant growth and development as an essential nutrient and second messenger. MCA1 is a plasma membrane protein and identified as a putative Ca^2+^ permeable mechanosensitive channel in plants [28,31]. A previous study showed that Ca^2+^ uptake is increased in rice *OsMCA*-overexpressing suspension-cultured cells [31], suggesting that *MCA1/DMT1* plays a role during Ca^2+^ transport in plants. In *Arabidopsis*, *AtMCA1* and *AtMCA2* have distinct and overlapping roles in Ca^2+^ uptake in roots [40]. Additionally, *NtMAC1* and *NtMAC2* partially complemented Ca^2+^ uptake defects of yeast mutants, which lacked mechanosensitive Ca^2+^ channel components [41]. Recently, *MCA1* and *MCA2* were shown to be involved in a cold-induced increase in Ca^2+^ in *Arabidopsis*, and cold-induced Ca^2+^ in *mca1* and *mca2* mutants was markedly lower than that in the WT [42]. The present study revealed that Ca^2+^ concentration was significantly higher in the *dmt1* mutant than in the WT. Apart from Ca^2+^, the levels of Mg^2+^, Mn^2+^ and Zn^2+^ were increased in the *dmt1* mutant (Table 1), suggesting that *DMT1* contributed to ion homeostasis in rice.

In eukaryotic cells, Ca^2+^ serves as a crucial mediator, whose intracellular concentration is tightly regulated by Ca^2+^ sensors in response to hormonal and environmental signals, including biotic and abiotic stresses. Here, we analyzed the expression patterns of the three largest categories of EF-hand proteins including CaMs (calmodulins), CMLs (CaM-like proteins) and CDPKs (calcium-dependent protein kinases) with transcriptome and proteome profiles. Several *OsCML* transcripts were significantly changed in the *dmt1* mutant compared to the WT, such as *OsCML2, OsCML11, OsCML15*, whereas all five *OsCaMs* were not significantly different in the *dmt1* mutant *(*Figure 5a–c). Previous studies showed that plants evolved a greatly expanded group of unique CMLs not found in animals, several CMLs have been proven to be Ca^2+^ sensors in response to biotic and abiotic stress in plants, such as *AtCML24* and *AtCML43* [22,43]. In the present study, several proteins were also detected in the *dmt1* mutant and the WT with iTRAQ in the proteome database, such as OsCML7, OsCML16, OsCaM1-3 and OsCBL6. Generally, protein level change trends were consistent with transcript expression levels. The present results suggested that *OsCMLs* might be involved in Ca^2+^ signal transducers in the *dmt1* mutant.

In conclusion, the present study revealed changes in gene and protein expression levels in the *dmt1* mutant and the WT. A series of differentially expressed factors, including GA and SL biosynthesis, and the presence of Ca^2+^ sensor genes were identified, and Ca^2+^ accumulated in the *dmt1* mutant. However, we have very limited knowledge of the mechanism underlying the plant architecture and Ca^2+^ uptake in the *dmt1* mutant. Additional studies will be needed to better understand the regulatory role of *DMT1* in ion homeostasis and plant architecture.

## 4. Materials and Methods

### 4.1. Plant Materials and Growth Conditions

The rice *japonica* cultivar Zhonghua 11 (ZH11) was used as the wild type in this study. The *dmt1* mutant was obtained from a T-DNA insertion line with a ZH11 background from the Shanghai Institute of Plant Physiology and Ecology, Chinese Academy of Sciences [44]. All rice plants were grown in the experimental field of South China Agricultural University in Guangzhou, southern China. Agronomic characters were recorded during the natural growing seasons. For laboratory work, rice seeds were soaked in ddH_2_O for 24 h, transferred to Kimura B nutrient solution (36.60 μM KNO_3_, 73.11 μM Ca(NO_3_)_2_·4H_2_O, 109.55 μM MgSO_4_, 18.25 μM K_2_SO_4_, 72.95 μM (NH_4_)_2_SO_4_, 36.45 μM KH_2_PO_4_, 4.01 μM FeSO_4_, 4.43 μM Na_2_EDTA, 1.82 μM MnCl_2_, 0.15 μM ZnSO_4_, 0.06 μM CuSO_4_, 9.26 μM H_3_BO_3_, 0.03 μM (NH_4_)_6_Mo_7_O_24_), and grown in a climate chamber (Hongrun, Nanjing, China) under a 16 h light/8 h dark cycle with the given light intensity (1000 mmol m^−2^s^−1^) at 28 °C. 

### 4.2. Analysis of the T-DNA Insertion Locus in dmt1 Mutant 

Inverse polymerase chain reaction (IPCR) was used to isolate the flanking sequence of T-DNA. Nested primers of the T-DNA right border primers were C1 and C2, and those of the left border primers were H1 and H2 (Figure S2). Initially, the genomic DNA was digested by HindIII. Primers for testing of the T-DNA inserting locus were 46490+ and 5TF1 for the left site and 47295- and 5TR1 for the right. Primers sequences are listed in Supplementary Table S1.

### 4.3. Gibberellin Treatment

Ten-day-old rice *dmt1* mutant and WT seedlings were sprayed with either 0 μM or 10 μM gibberellin (GA_3_, Solarbio, Beijing, China) three times every 2 days. The lengths of shoots were recorded on the 7th day after the first treatment.

### 4.4. RNA Sequencing

Leaves of the WT and *dmt1* mutant during the tillering stage were harvested and preserved in liquid nitrogen and stored at −80 °C. TRIzol™ (Invitrogen™, Waltham, America) reagent was used to extract the total RNA of samples according to the manufacturer’s instructions. Three replicates were prepared for both WT and *dmt1* mutant, approximately 0.5 g leaves used in each replication. The cDNA library was constructed by Illumian Truseq RNA sample prep Kit, and sequencing by Illumina HiSeq 2500 (Illumina, San Diego, America). Raw data (raw reads) in fastq format were firstly processed through in-house perl scripts. In this step, the clean data (clean reads) were obtained by removing reads containing adapters, poly-N, and low-quality reads from the raw data. At the same time, quality parameters of the clean data, including Q20, Q30, GC content, and sequence duplication level were used for data filtering. All the succeeding analyses were conducted using high-quality clean data. Reference genome and gene model annotation files were downloaded from The MSU Rice Genome Annotation Project Database website at http://rice.plantbiology.msu.edu/. An index of the reference genome was built using Bowtie2 v2.2.5 and paired-end clean reads were aligned to the reference genome using TopHat v2.0.14. TopHat was chosen as the mapping tool because it can generate a database of splice junctions based on the gene model annotation file, and thus, provided better mapping results than other non-splice mapping tools. All raw data have been deposited in the Sequence Read Archive data repository (accession PRJNA602017) of the NCBI.

### 4.5. Protein Preparation and Digestion 

The iTRAQ assay was performed by the BGI Company. For each of three biological replicates of WT and *dmt1* mutants, proteins were extracted from leaves in the tillering stage and approximately 1 g leaves used in each replication. Initially, leaf samples were ground into powder in liquid nitrogen, extracted with Lysis buffer (7 M Urea, 2 M Thiourea, 4% CHAPS, 40 mM Tris-HCl, pH 8.5) containing 1 mM PMSF and 2 mM EDTA (final concentration). After 5 min, 10 mM DTT (final concentration) was added to the samples. The suspension was sonicated at 200 W for 15 min and then centrifuged at 4 °C, 30,000× *g* for 15 min. The supernatant was mixed well with 5 × volume of chilled acetone containing 10% (*v/v*) TCA and incubated at -20 °C overnight. After centrifugation at 4 °C, 30,000× *g*, the supernatant was discarded. The precipitate was washed with chilled acetone three times. The pellet was air-dried and dissolved in Lysis buffer (7 M urea, 2 M thiourea, 4% NP40, 20mM Tris-HCl, pH 8.0–8.5). The suspension was sonicated at 200 W for 15 min and centrifuged at 4 °C, 30,000× *g* for 15 min. The supernatant was transferred to another tube. To reduce disulfide bonds in proteins of the supernatant, 10 mM DTT (final concentration) was added and incubated at 56 °C for 1 h. Subsequently, 55 mM IAM (final concentration) was added to block the cysteines, incubated for 1 h in the darkroom. The supernatant was mixed well with 55 × volume of chilled acetone for 2 h at −20 °C to precipitate proteins. After centrifugation at 4 °C, 30 000× *g*, the supernatant was discarded, and the pellet was air-dried for 5 min, dissolved in 500 μL 0.5 M TEAB (Applied Biosystems, Milan, Italy), and sonicated at 200 W for 15 min. Finally, samples were centrifuged at 4 °C, 30,000× *g* for 15 min. The supernatant was transferred to a new tube and quantified. The proteins in the supernatant were kept at −80 °C for further analysis. The proteins were quantified by Bradford method.

Total protein (100 μg) was taken out of each sample solution and then the protein was digested with Trypsin Gold (Promega, Madison, WI, USA) with the ratio of protein:trypsin = 30:1 at 37 °C for 16 h. After trypsin digestion, peptides were dried by vacuum centrifugation. Peptides were reconstituted in 0.5 M TEAB and processed according to the manufacture’s protocol for 8-plex iTRAQ reagent (Applied Biosystems, Waltham, America). Briefly, one unit of iTRAQ reagent was thawed and reconstituted in 24 μL isopropanol. The peptides were labeled with the isobaric tags, incubated at room temperature for 2 h. The labeled peptide mixtures were then pooled and dried by vacuum centrifugation. SCX chromatography was performed with an LC-20AB HPLC Pump system (Shimadzu, Kyoto, Japan). The iTRAQ-labeled peptide mixtures were reconstituted with 4 mL buffer A and loaded onto a 4.6 × 250 mm Ultremex SCX column containing 5 μm particles (Phenomenex). The peptides were eluted at a flow rate of 1 mL/min with a gradient of buffer A (25 mM NaH_2_PO_4_ in 25% ACN, pH 2.7) for 10 min, 5–60% buffer B for 27 min, 60–100% buffer B for 1 min. The system was then maintained in 100% buffer B (25mM NaH_2_PO_4_, 1 M KCl in 25% ACN, pH 2.7) for 1 min before equilibrating with buffer A for 10 min prior to the next injection. Elution was monitored by measuring the absorbance at 214 nm, and fractions were collected every 1 min. The eluted peptides were pooled into 20 fractions, desalted with a Strata X C18 column (Phenomenex) and vacuum dried.

### 4.6. LC-ESI-MS/MS Analysis Based on Triple TOF 5600

Each fraction was resuspended in buffer A (5% ACN, 0.1%FA) and centrifuged at 20,000× *g* for 10 min, the final concentration of peptide was about 0.5 μg/μL on average. 10 μL supernatant was loaded on a LC-20AD nanoHPLC (Shimadzu, Kyoto, Japan) by the autosampler onto a 2 cm C18 trap column. Then, the peptides were eluted onto a 10cm analytical C18 column (inner diameter 75 μm) packed in-house. The samples were loaded at 8 μL/min for 4min, then the 35min gradient was run at 300 nL/min starting from 2 to 35% B (95% ACN, 0.1% FA), followed by 5 min linear gradient to 60%, then, followed by 2 min linear gradient to 80%, and maintenance at 80% B for 4 min, and finally return to 5% in 1 min. Data acquisition was performed with a Triple TOF 5600 System (AB SCIEX, Concord, ON) fitted with a Nanospray III source (AB SCIEX, Concord, ON) and a pulled quartz tip as the emitter (New Objectives, Woburn, MA). Data were acquired using an ion spray voltage of 2.5 kV, curtain gas at 30 psi, nebulizer gas at 15 psi, and an interface temperature of 150 °C. The MS was operated with an RP ≥ 30 000 FWHM for TOF MS scans. For IDA, survey scans were acquired in 250 ms and as many as 30 product ion scans were collected if exceeding a threshold of 120 counts/s and with a 2+ to 5+ charge state. The total cycle time was fixed to 3.3 s. The Q2 transmission window was 100 Da for 100%. Four time bins were summed for each scan at a pulser frequency value of 11 kHz by monitoring the 40 GHz multichannel TDC detector with four-anode channel detection ions. A sweeping collision energy setting of 35 ± 5 eV coupled with iTRAQ adjusted to a rolling collision energy was applied to all precursor ions for collision-induced dissociation. Dynamic exclusion was set for 1/2 of peak width (15 s), and then the precursor was refreshed off the exclusion list.

### 4.7. Proteomic Data Analysis

The protein identification was performed using a Mascot search engine (Matrix Science, London, UK; version 2.3.02). For protein quantitation, it was required that a protein contained at least two unique spectra. The quantitative protein ratios were weighted and normalized by the median ratio in Mascot. We only used ratios with *p*-values < 0.05, and only fold changes of > 1.5 were considered to be a significant change. Functional annotations of the proteins were conducted using Blast2GO program against the non-redundant protein database (NR; NCBI). The KEGG database (http://www.genome.jp/kegg/) and the COG database (http://www.ncbi.nlm.nih.gov/COG/) were used to classify and group these identified proteins. The mass spectrometry proteomics data have been deposited in ProteomeXchange (submission No. PXD017197). Reviewer account details for ProteomeXchange (Username: reviewer04292@ebi.ac.uk; Password: 4FopczCR).

### 4.8. Expression Analysis by Real-Time PCR

Total RNA was extracted from different samples using TRIzol™ reagent (Invitrogen™, Waltham, America), Reverse transcription (RT) was performed using 5 × All-In-One RT MasterMix (abm^®^, Vancouver, Canada) according to manufacturer’s instruction. Quantitative real-time polymerase chain reaction (qRT-PCR) was performed as previously described [44]. The relative expression levels of target genes were normalized to that of rice *ACTIN1*. Real-time PCR gene expression data from three independent biological replicates. All primers used in qRT-PCR are listed in Supplemental Table S1.

### 4.9. Elemental Analysis by Inductively Coupled Plasma Optical Emission Spectrometer (ICP-OES)

Tissues were harvested during the tillering stage and dried in a 60 °C oven for 48 h, then the dried tissues were predigested overnight in borosilicate glass tubes with 4 mL of redistilled 98.8% HNO_3_. One milliliter of concentrated trace metal grade HClO_4_ was added to the predigested tissues and heated to 100 °C for 1 h, 150 °C for 1 h, 180 °C for 1 h, and then 210 °C to dryness (1–2 h). Digestions were performed using a heating block with an exhaust-collecting manifold. Digests were resuspended in 15 mL redistilled 2% HNO_3_. Concentrations of Al, Ca, K, Mg, P, Fe, Cu, Mn, and Zn were determined by inductively coupled plasma-optical emission spectroscopy. Element concentrations were expressed as mg/g dry weight ($C=\frac{c\cdot V}{DW}$, c was element concentrations as measured by ICP-OES (Varian, California, America), V= 15 mL, and DW= 1 g).

## Figures and Tables

**Table 1:** Several ions accumulate in the *dmt1* mutant and the WT at the tilling stage.

Elements	Concentrations (mg/g)	
	**WT**	***dmt1***
Al	0.147 ± 0.013	0.168 ± 0.012
Ca	2.730 ± 0.039	5.091 ± 0.069 **
K	54.690 ± 1.838	52.945 ± 0.177
Mg	1.578 ± 0.342	3.435 ± 0.115 **
P	5.353 ± 0.095	6.393 ± 0.260 **
Cu	0.030 ± 0.011	0.055 ± 0.005
Fe	0.180 ± 0.021	0.310 ± 0.006 **
Mn	0.866 ± 0.103	1.395 ± 0.127 *
Zn	0.068 ± 0.004	0.143 ± 0.029 *

Values are the means ± SE. Asterisks represent a significant difference between *dmt1* and the WT. * *p* < 0.05, ** *p* < 0.01, Student’s *t*-test.

## Supplementary Materials

Supplementary materials can be found at https://www.mdpi.com/1422-0067/21/3/1097/s1.
